# HOW DOES MEDICARE ELIGIBILITY AFFECT CHOICE AND UTILIZATION?: EVIDENCE FROM VETERANS

**DOI:** 10.1093/geroni/igae098.2456

**Published:** 2024-12-31

**Authors:** Marion Aouad, Liam Rose, Todd Wagner

**Affiliations:** University of California Irvine, Irvine, California, United States; Stanford University, Stanford, California, United States; Stanford University, Stanford, California, United States

## Abstract

Health insurance streamlines access to care, thereby improving well-being and health. In efforts to control costs, health insurers use selective contracting to limit the size of their provider networks. Regulators have focused on network adequacy rules, given concerns that overly restrictive networks may impede appropriate access. This raises an important question about what happens to patient behavior if a network expanded. We examine the effects of network expansions by focusing on veterans who receive care from the Veterans Health Administration (VHA). We exploit the change in their insurance network induced by Medicare eligibility at age 65 to examine changes in inpatient health care behaviors. Using regression discontinuity methods, we find that hospitalizations increase by approximately 3 percent at 65. Interestingly, there are large insurer reallocations – individuals increasingly choose the new Medicare option for hospitalizations once it is available (a 70 percent increase relative to the pre-65 share.) while the VHA and commercial insurers are used increasingly less, with an approximately 7 and 51 percent decline, compared to the pre-65 share, respectively. We also find larger reallocations toward Medicare for certain groups, particularly among individuals who live closest to a Medicare hospital and further from VHA hospitals, and among those who visit the hospital for elective medical procedures (e.g., knee replacement). The results show the link between network size and cost containment, with expansions resulting in greater use of elective inpatient care.

